# National Conference of State Medical Examining and Licensing Boards

**Published:** 1894-05

**Authors:** 


### NATIONAL CONFERENCE OF STATE MEDICAL EXAMINING AND LICENSING BOARDS.

THE next meeting of this body will be held in San Francisco, June 5, 1894. The important object of this association should commend itself to the friends of higher medical education all over the land. As the years advance, the number of states that require separate license is increasing, and we predict that it will not be long before every commonwealth in the Union will be governed by such laws.

The object of this national conference, now about to hold its fourth meeting, is to unify and standardize the methods in vogue in the several states, and to otherwise improve the service.

The death of Dr. John H. Rauch, president of the conference, is a sad blow to the cause to which he gave so many years of his life. The Bulletin of the American Academy of Medicine justly says that he was one of the best friends the profession of medicine in this country ever had, and that his name will be held in honor wherever the history of American medicine is studied.

Dr. Charles K. Cole, of Helena, Montana, secretary of the conference, has prepared and sent out a circular containing the following inquiries :

How long has your board been organized under your law ?

During that period how many candidates have been examined, and what percentage of those examined have passed and received license to practice ? (Note : The above should be separated into years, if possible.)

How is your board constituted with reference to the various "Schools of Practice ?"

Do you favor the plan of mixed boards, or three separate boards ?

How many members should constitute a state board, and what is the desirable appointing power ?

Should teachers in medical schools be eligible to membership on state examining boards ?

Does your board look with favor upon the idea of interchange of certificates between state boards?

What, in your opinion, are the leading defects in your law, and in the laws of the various states?

What should constitute a "college in good standing" with reference to number of years of study required and curriculum, for purpose of registration.

Upon what points in your medical practice act (if any) have you had litigation in the courts, and what has been the result of such litigation?

How many practitioners are there in your state (number in each school of practice if convenient, number of army and navy surgeons and surgeons of the Marine Hospital service), and how many are practising illegally? (Note: This should include midwives.)

Will you kindly send the secretary a copy of your law for the use of the conference?

The attention of officers and members of the State Medical Examining and Licensing Boards is called to these questions, and it is requested that answers thereto be sent to Dr. Cole at the earliest possible day. The facts and answers thus obtained will be carefully tabulated, and presented to the conference for consideration.

